# The development of guidelines for the early recognition of foot melanoma for Podiatrists

**DOI:** 10.1186/1757-1146-3-S1-O5

**Published:** 2010-12-20

**Authors:** Ivan Bristow, David de Berker

**Affiliations:** 1 School of Health Sciences, University of Southampton, Southampton, UK; 2 Dermatology Department, Bristol Royal Infirmary, Bristol, UK

## Introduction

The incidence of malignant melanoma (MM) continues to rise in the UK and Europe. Despite being a common form of skin cancer it is responsible for the majority of skin cancer related deaths. Around 3-15% of all cutaneous MM arise on the foot. However, lesions arising on the foot hold a poorer prognosis than melanoma elsewhere due to frequent misdiagnosis and late presentation. Initiatives to raise practitioner awareness to the problem would hopefully facilitate earlier referral and diagnosis.

## Methods

Following discussions with the Faculty of Podiatric Medicine and General Practice, a panel including Dermatologists (with an interest in skin cancer) and a Podiatrist was assembled and undertook a project to produce a guide for Chiropodists/Podiatrists on the early recognition of malignant melanoma arising on the foot.

## Results

Following a systematic review of published evidence and existing guidelines it was concluded there was insufficient research to fully inform such a guide. Therefore using the available literature and case reports a paper was drafted using expert opinion and clinical experience. Evaluation of reported cases of misdiagnosis and clinical features lead to the development of a new acronym specific to foot malignancy with clinical information and suggested actions. In addition, specific advice has been drafted on nail melanoma highlighting common clinical features and differential diagnosis.

## Conclusion

The guide is currently being field tested amongst practitioners but should ultimately be available to all Podiatrists and Chiropodists to help raise awareness of this aggressive and often fatal disease.

